# Intraoperative Asystole during Thoracoscopic Left Lung Upper Lobectomy: A Case Report

**DOI:** 10.70352/scrj.cr.25-0576

**Published:** 2025-11-13

**Authors:** Yu Sugimoto, Masatoshi Kanayama, Misono Kobayashi, Natsumasa Nishizawa, Yasuhiro Chikaishi, Manabu Yasuda, Fumihiro Tanaka

**Affiliations:** 1 Department of Chest Surgery, Iizuka Hospital, Iizuka, Fukuoka, Japan; 2 2nd Department of Surgery, School of Medicine, University of Occupational and Environmental Health, Japan, Kitakyushu, Fukuoka, Japan

**Keywords:** intraoperative asystole, lobectomy, lung cancer

## Abstract

**INTRODUCTION:**

Intraoperative asystole is a rare but potentially life-threatening complication of lung cancer surgery. Various factors, including cardiac conditions, hemorrhage, anesthetic effects, and neural reflexes, may contribute to this phenomenon. Herein, we report a case of intraoperative asystole triggered by a vagal reflex during thoracoscopic left upper lobectomy.

**CASE PRESENTATION:**

A 53-year-old man with left upper lobe adenocarcinoma of the lung (cT1cN0M0, Stage IA3) underwent video-assisted thoracoscopic surgery. During blunt dissection along the posterior surface of the left superior pulmonary vein, severe bradycardia rapidly progressed to asystole. Surgical manipulation was discontinued immediately, and spontaneous circulation returned within 40 s without pharmacological intervention. As the event resolved immediately upon cessation of surgical stimulation, it was considered a transient reflex response. The surgery was resumed and completed without complications. The patient’s postoperative course was uneventful.

**CONCLUSIONS:**

Although rare, vagal reflex–induced asystole should be considered during manipulation of the left pulmonary hilum. Therefore, surgeons and anesthesiologists must be vigilant and prepared for immediate resuscitative measures to ensure patient safety.

## Abbreviation

VATS
: video-assisted thoracoscopic surgery

## INTRODUCTION

Intraoperative asystole during lung cancer surgery is a rare but potentially life-threatening complication. This event can be triggered by complex factors, including cardiac causes, massive hemorrhage, hypoxia, acidosis, electrolyte disturbances, anesthetic effects (e.g., systemic or epidural anesthesia), and thoracic irrigation with hypotonic solutions, such as distilled water.^1)^ Although uncommon, nerve reflex-mediated arrhythmias may occur abruptly and require immediate recognition and intervention.^2)^ Therefore, intraoperative teams must remain vigilant and prepared to respond promptly and decisively to emergencies.

Herein, we report a case of sudden asystole that occurred during thoracoscopic left upper lobectomy, specifically during blunt dissection around the left superior pulmonary vein. We examined the potential pathophysiological mechanisms of this phenomenon and reviewed relevant literature to provide practical insights into intraoperative risk mitigation and emergency response strategies.

## CASE PRESENTATION

**Fig. 1:**
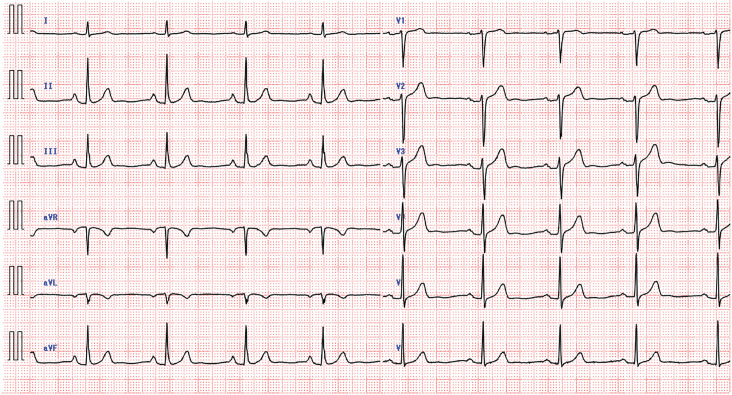
Twelve-lead electrocardiogram showing a normal sinus rhythm at 65 bpm, with normal QRS morphology and axis, and no apparent conduction delay or significant ST-segment or T-wave abnormalities. bpm, beats per minute

**Fig. 2:**
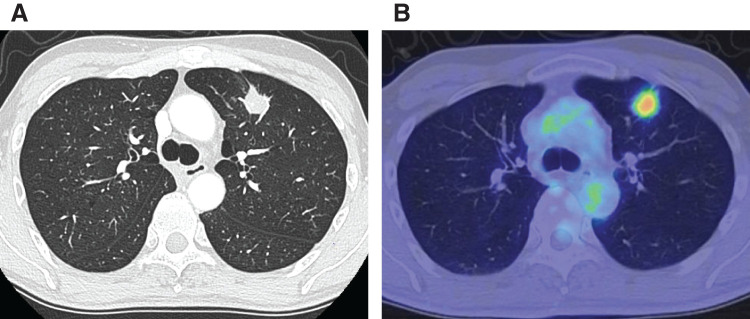
(**A**) Chest CT showing a 23 × 20-mm nodule in the upper lobe of the left lung. (**B**) PET-CT showing increased fluorodeoxyglucose uptake in the same lesion (maximum standardized uptake value, 4.89).

A 53-year-old man with no history of cardiac disease was referred to our hospital after an incidental pulmonary nodule was detected in the upper lobe of the left lung on CT performed during an evaluation of an unrelated illness. Preoperative 12-lead electrocardiogram results were within normal limits (**Fig. 1**). The patient was asymptomatic. Chest radiography revealed no abnormalities. Contrast-enhanced CT revealed a 23 × 20-mm nodule located in segment S3 of the left upper lobe of the lung (**Fig. 2A**). PET-CT demonstrated increased fluorodeoxyglucose uptake in the same region, with a maximum standardized uptake value of 4.89 (**Fig. 2B**). No evidence of lymph node or distant metastasis was observed. Transbronchial lung biopsy confirmed a diagnosis of adenocarcinoma (cT1cN0M0, Stage IA3). VATS with systemic lymph node dissection (ND2a-1) was planned for the left upper lobectomy.

**Fig. 3:**
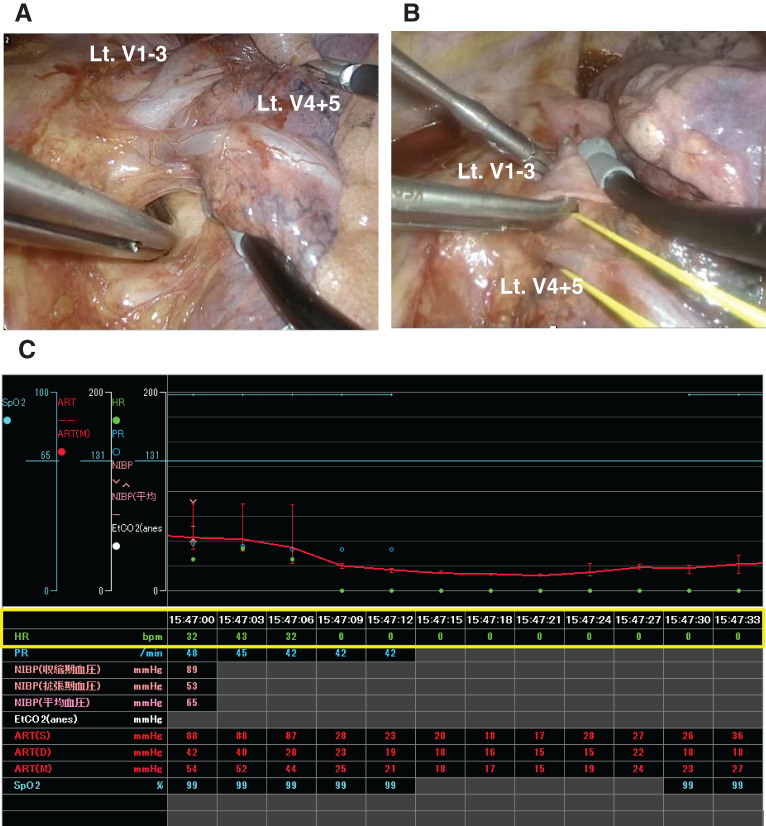
(**A**) Blunt dissection along the posterior surface of the left superior pulmonary vein (V1–3 and V4 + 5). (**B**) Intraoperative view at the onset of bradycardia during dissection near the posterior aspect of the vein. (**C**) Intraoperative monitor showing sudden bradycardia progressing to transient asystole. Heart rate dropped to 0 bpm and recovered spontaneously within 40 s. bpm, eats per minute; Lt, left

The procedure was performed under general anesthesia using 1-lung ventilation with the patient in the right lateral decubitus position. Three ports were created: a 4.0-cm incision in the 4th intercostal space at the anterior axillary line; a 2.0-cm incision in the 6th intercostal space at the level of the inferior scapular angle; and a 1.5-cm incision in the 7th intercostal space at the mid-axillary line. Following the division of the interlobar arteries A4 + 5, the posterior portion of the major fissure, and A1 + 2c, hilar dissection of the left superior pulmonary vein was performed. Blunt dissection was performed along the vein, particularly on the posterior surface (**Fig. 3A**). Although we intended to tape the vein en bloc (V1–3 and V4+5), the anatomical relationships and firm perivascular tissue around V1–3 made this technically difficult. Therefore, V4+5 was taped first, followed by continued dissection along the posterior aspect of V1–3. While attempting to pass a right-angle dissector behind the vein to place a vessel loop, the patient suddenly developed bradycardia that rapidly progressed to asystole (**Fig. 3B** and **3C**). Surgical manipulation was stopped immediately, and preparations for emergency thoracotomy were initiated with the intent of performing an open cardiac massage. However, spontaneous cardiac rhythm returned within approximately 40 s without the use of vasopressors or other circulatory agents. As the event resolved immediately upon cessation of surgical stimulation, it was considered a transient reflex response.

The operation was resumed with extreme caution. We avoided the use of energy devices around the pulmonary hilum, minimized traction on the left superior pulmonary vein and adjacent structures, and proceeded with meticulous sharp dissection. With these precautions, the left upper lobectomy was completed without further incidents. Postoperatively, the patient underwent continuous electrocardiographic monitoring, which revealed no abnormalities. The chest drain was removed on POD 2, and the patient was discharged uneventfully on POD 6.

## DISCUSSION

In the present case, asystole occurred during blunt dissection around the posterior aspect of the left superior pulmonary vein without the use of an energy device. A literature review identified 9 cases,^3–8)^ including ours, in which intraoperative cardiac arrest occurred during lung cancer surgery in association with surgical manipulation (**Table 1**). Overall, intraoperative asystole rarely occurs; however, it may occasionally be encountered in daily clinical practice. Nevertheless, these events have rarely been systematically summarized or analyzed in the literature. Our report highlights an under-recognized but clinically important phenomenon that warrants further investigation.

**Table 1:** Summary of reported cases of intraoperative cardiac arrest during lung cancer surgery attributed to intrathoracic manipulation

Case	Age/sex	Cardiac history	Surgical procedure	Approach	Triggering maneuver	Anatomical site involved	Intervention for cardiac arrest	Arrest time (s)	Epidural anesthetics	Recurrent event during surgery	Reference
1	61/M	HT	LUL+ND2a-1	Thoracotomy	Electrocautery	#5 L/N	Open cardiac massage, ephedrine, atropine	15	+ (Th6–7)	−	Yamamoto et al.^3)^
2	67/M	HT	LLL+ND2a	Thoracotomy	Electrocautery	Hilar mediastinal pleura	Open cardiac massage, epinephrine, ephedrine, lidocaine	※	+ (Th6–7)	−	Watari and Matsuura^4)^
3	71/M	—	LUL+ND2a	Thoracotomy	Electrocautery	Hilar mediastinal pleura	Open cardiac massage, epinephrine, ephedrine, atropine	※	−	−	Watari and Matsuura^4)^
4	66/M	—	LUL+ND2a	Thoracotomy	Electrocautery	Hilar mediastinal pleura	Open cardiac massage, ephedrine, atropine	※	+ (Th6–7)	−	Watari and Matsuura^4)^
5	69/F	HT	LUL+ND2a-1	VATS	Vessel sealing device/traction	Hilar portion of PA	Cardiac massage via a thoracoscopic port	60	−	+	Kawana et al.^5)^
6	59/F	—	LUL+ND2a-1	VATS	Ultrasonic surgical device	#5, 6 L/N	Cardiac massage via a thoracoscopic port	20	+ (Th6–7)	−	Iwai et al.^6)^
7	55/M	—	LUL+ND2a	Thoracotomy	Electrocautery	#5 L/N	Open cardiac massage, epinephrine	60	−	+	Lee et al.^7)^
8	68/M	HT, hypertrophic cardiomyopathy	Exploratory thoracotomy	Thoracotomy	Not described	#5 L/N	Open cardiac massage, ephedrine	240	+ (Th8–9)	−	Sakai et al.^8)^
9	53/M	—	LUL+ND2a-1	VATS	Traction	Hilar portion of PV	Surgical interruption only	40	−	−	Our case

^※^ Cardiac rhythm resumed promptly following cardiac massage.

F, female; HT, hypertension; L/N, lymph node; LLL, left lower lobectomy; LUL, left upper lobectomy; M, male; ND, nodal dissection; PA, pulmonary artery; PV, pulmonary vein; Th, thoracic vertebrae; VATS, video-assisted thoracoscopic surgery

**Fig. 4:**
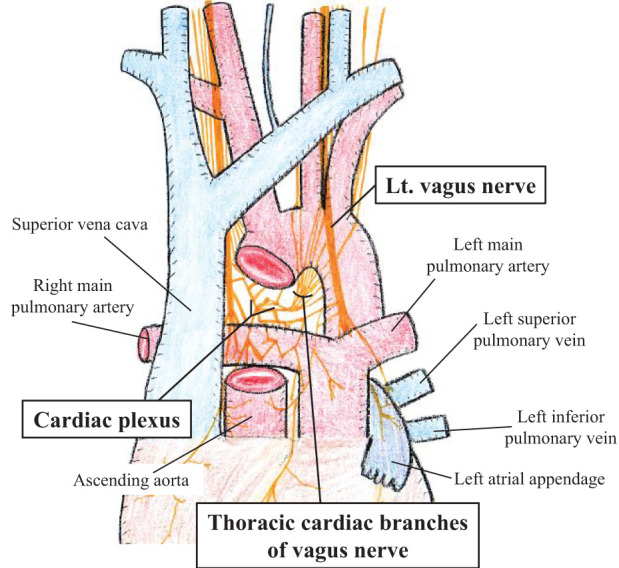
Anatomy of the cardiac plexus. The illustration shows the anatomical relationship between the cardiac plexus, vagal branches, and surrounding thoracic structures. Lt., left

Notably, all of these events involved the left pulmonary hilum or adjacent mediastinal structures, where the vagal branches and cardiac plexus are densely distributed^9)^ (**Fig. 4**). Triggers of neural stimulation include electrical sources (e.g., electrocautery and ultrasonic devices) and mechanical factors (e.g., traction and vascular taping). Although most previously reported cases have implicated electrical stimulation, our case is unique in that asystole resulted solely from mechanical manipulation. During the tunneling of V1–3, the posterior aspect of the left superior pulmonary vein and adjacent vagal branches were subjected to transient traction and compression by surgical instruments, despite the absence of energy device use. Such maneuvers likely stimulated the perivascular autonomic cardiac plexus, leading to a profound vagal reflex and subsequent asystole. Similar events have been reported in non-thoracic surgeries,^10,11)^ suggesting that excessive traction near autonomic cardiac innervation may be sufficient to induce profound vagal responses. Furthermore, case 5 demonstrated recurrent bradycardia during blunt dissection following an initial episode triggered by electrocautery, indicating that mechanical stimulation alone can provoke vagal reflexes. These findings emphasize the need for caution not only during energy device use but also when performing dissection or traction near autonomic neural structures.

Immediate cessation of surgical manipulation is critical for bradycardia or asystole. In our case, sinus rhythm spontaneously returned during preparation for thoracotomy. In nearly all the reported cases, prompt resuscitative measures, including cardiac massage and pharmacological intervention, led to rapid recovery. In case 8, sinus rhythm was restored after 240 s, possibly because of pre-existing cardiac disease, underscoring the importance of a rapid, coordinated response by the surgical and anesthetic teams.

A post hoc review of the intraoperative monitoring records revealed a single brief episode of bradycardia (30–40 bpm) beginning about 15 s before the onset of asystole. Earlier recognition of this evolving bradyarrhythmia through closer surgeon-anesthesiologist coordination might have enabled timely intervention and prevented progression to asystole.

In thoracoscopic surgery, immediate manual cardiac massage is often technically challenging. In case 5, compression through the thoracoscopic port was successfully initiated and contributed to patient stabilization. If the initial measures fail, prompt conversion to thoracotomy should be performed. As minimally invasive techniques, such as uniportal VATS and robotic surgery, have become more widespread, preparedness for emergency thoracotomy and resuscitation has become increasingly essential. In most cases of early rhythm recovery, surgery was completed without recurrence, and the postoperative outcomes were favorable. This finding suggests that vagally mediated asystole in this context is typically transient and reversible. However, as demonstrated in case 8, delayed rhythm recovery or repeated bradycardia may require staged surgery under temporary pacing. Although epidural anesthesia has been suggested as a contributing factor,^12,13)^ several cases, including ours, occurred without its use. Furthermore, except for 1 case of hypertrophic cardiomyopathy, most patients had no significant cardiac comorbidities. These findings support the conclusion that surgical manipulation is a primary precipitating factor, particularly in anatomically vulnerable areas. Therefore, special attention should be paid during procedures involving the upper lobe of the left lung, where the vagus nerve and cardiac plexus are at risk. A thorough understanding of the regional anatomy and careful technique when using energy devices or applying traction are essential. If intraoperative bradycardia or asystole occurs, the surgical procedure should be stopped immediately and intravenous atropine sulfate should be considered. The use of atropine is indeed recommended when bradycardia occurs during surgery, and effective communication and coordination between the surgeon and anesthesiologist are essential to prevent asystole.

## CONCLUSIONS

Although rare, vagal reflex-induced asystole during manipulation of the left pulmonary hilum carries a significant risk and can be fatal if not properly recognized and managed. Thoracic surgeons and anesthesiologists must be vigilant and prepared to respond swiftly to ensure patient safety.

## DECLARATIONS

### Availability of data and materials

In this study, no data were generated or analyzed; therefore, data sharing is not applicable.
